# How lung cancer cells change identity

**DOI:** 10.7554/eLife.71610

**Published:** 2021-08-04

**Authors:** Mitchell S von Itzstein, Benjamin J Drapkin, John D Minna

**Affiliations:** 1 Department of Internal Medicine, Division of Hematology and Oncology, the Hamon Center for Therapeutic Oncology Research, University of Texas Southwestern Medical Center Dallas United States; 2 Simmons Comprehensive Cancer Center, University of Texas Southwestern Medical Center Dallas United States

**Keywords:** lung cancer, lineage transformation, ERK signaling, lineage switching, Human

## Abstract

Changes in MAPK signaling allow lung cancer cells to transition between lineages that respond differently to treatment.

**Related research article** Inoue Y, Nikolic A, Farnsworth D, Shi R, Johnson FD, Liu A, Ladanyi M, Somwar R, Gallo M, Lockwood WW. 2021. Extracellular signal-regulated kinase mediates chromatin rewiring and lineage transformation in lung cancer. *eLife* **10**:e66524. doi: 10.7554/eLife.66524

Cancer cells that arise from different areas of the body will share several common hallmarks (Hanahan and Weinberg, 2011). However, it is possible to distinguish between these cells as they will also retain features of their tissue of origin. This lineage is important because it helps determine which cancer treatments will be the most effective and whether the genes responsible for making the cell cancerous can be targeted with a specific therapy.

However, some cancer cells can also switch lineage, allowing them to evade treatments tailored to these specific ‘oncogenes’ (Quintanal-Villalonga et al., 2020). Reminiscent of the differentiation process that takes place for stem cells during embryonic development, this ability to transition from one lineage to another could be genetically or epigenetically encoded and may represent a hallmark of cancer (Le Magnen et al., 2018).

A clinically important example of lineage switching is seen in a type of lung cancer known as adenocarcinomas (or LUADs). Some of these tumors have mutations in the genes coding for either the EGFR or KRAS proteins, which overactivate the MAPK signaling pathway – a molecular cascade which normally helps cells to respond appropriately to their environment by controlling cell division and other biological processes.

LUADs with mutations in the gene for EGFR can be treated with a therapy that specifically targets this oncogene. However, after treatment about 5% of these LUADs will adopt another lineage, turning instead into an aggressive neuroendocrine tumor called small cell lung cancer (SCLC; Offin et al., 2019; Aggarwal et al., 2018). Neuroendocrine cancer can also emerge at other sites in the body, such as the prostate following treatment with another targeted therapy. Regardless of their origin, all advanced neuroendocrine tumors share similar molecular features, are difficult to treat and can spread quickly (Park et al., 2018). They also do not respond to the EGFR-targeted drugs used to treat LUADs, as they rarely express the mutated proteins, even if the mutations are present in the genome. Conversely, SCLCs that become resistant to standard chemotherapy may lose expression of neuroendocrine features and transition to another lineage. Although recent studies have shed light on how lineage switching happens at the molecular level (Marjanovic et al., 2020; Ku et al., 2017; Chen et al., 2019), there are still crucial gaps in our knowledge about the biological mechanisms underlying these transitions.

Now, in eLife, William Lockwood from the British Columbia Cancer Agency and co-workers in Canada and the United States – including Yusuke Inoue as first author – report that the MAPK signaling pathway may regulate lineage switching in lung cancer cells (Inoue et al., 2021). First, experiments revealed that activating MAPK signaling, by increasing the levels of EGFR and KRAS, caused SCLC cells from patients to express fewer neuroendocrine markers when cultured in the laboratory. In particular, MAPK signaling reduced the expression of transcription factors needed for neuroendocrine cell traits. This suggests that the high levels of MAPK activation normally found in LUADs usually prevents these cancer cells from adopting a neuroendocrine identity (Figure 1). Furthermore, these molecular changes impacted how the tumor cells grew and behaved, suggesting that these transformed SCLC cells can be used as a preclinical model for studying aspects of lineage switching.

**Figure 1.**
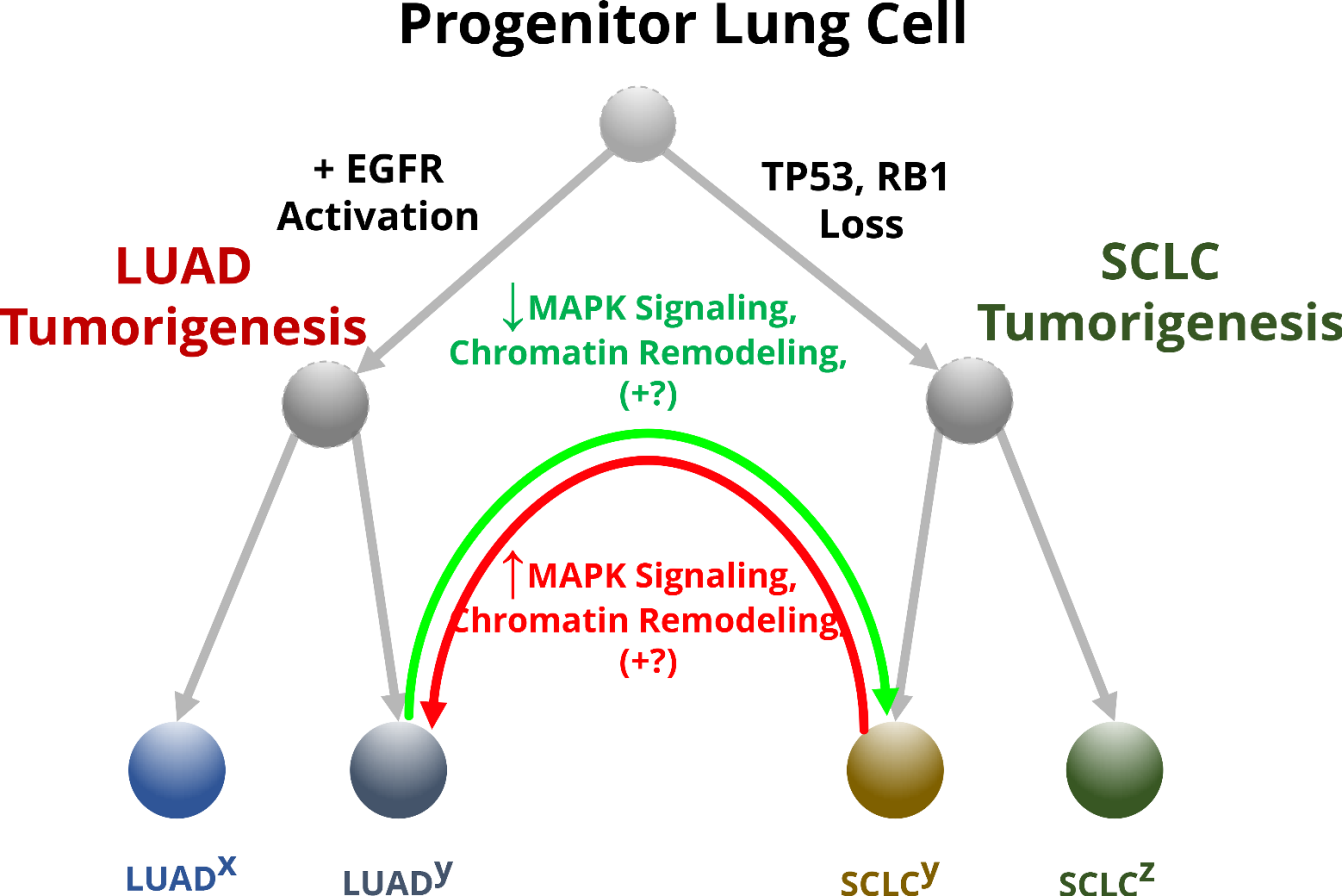
The effect of MAPK signaling on lineage switching in lung cancer cells. Progenitor lung cells are able to develop into different types of cancer. Cells with high levels of EGFR and other proteins involved in the MAPK pathway are more likely to transition in to lung adenocarcinomas (LUADs; left), while mutations that impair the tumor suppressor genes *TP53* and *RB1* lead to the formation of neuroendocrine tumors called small cell lung cancers (SCLCs; right). Some of these cells (annotated with a superscript ‘y’) will undergo further molecular changes that will allow them to transition between these two lineages, while others (annotated with superscripts ‘x’ and ‘z’) will be unable to change their identity. For example, LUADs with mutations that impair the activity of *TP53* and *RB1* can switch to a SCLC phenotype (green arrow). Inoue et al. found that this conversion depends on the reduced activity of the MAPK signaling pathway, plus additional unknown factors (indicated with +?): this alters the structure of chromatin so that the transcription machinery of the cell can access and switch on the genes for transcription factors that specify the neuroendocrine identity. Some SCLC cells can also transition into LUADs (red arrow) by reactivating the MAPK signaling pathway, which leads to chromatin remodeling that limits access to the genes for the neuroendocrine transcription factors.

Further experiments showed that the terminal component of the MAPK pathway, known as ERK, is responsible for changing the expression of transcription factors which specify neuroendocrine identity. Inoue et al. found that ERK activates two proteins (CBP and p300) that alter the conformation of the DNA segments in which the genes for the neuroendocrine transcription factors reside, stopping them from being transcribed. When ERK and CBP/p300 were inhibited, increasing the levels of EGFR and KRAS no longer reduced the expression of neuroendocrine transcription factors. This provides strong evidence that these three proteins (ERK, CBP and p300) all play a key role in controlling neuroendocrine identity.

Next, Inoue et al. attempted to recapitulate the lineage transition seen in patients, where LUADs occasionally switch to a neuroendocrine SCLC phenotype. To do this, they first genetically engineered LUAD cells with existing mutations in the gene for EGFR and the tumor suppressor gene *TP53* to have an additional molecular change which reduces the activity of another tumor suppressor gene called *RB1*: these genetic alterations are found in nearly all LUADs that assume a neuroendocrine phenotype as a resistance mechanism to EGFR-targeted therapy. These cells were then exposed to a potent EGFR inhibitor. However, despite the cells becoming resistant to the inhibitor treatment, no change in growth characteristics, lineage markers or switch to a neuroendocrine phenotype were identified.

It is unclear why this strategy did not work, and what additional steps are needed for LUADs to fully transition into neuroendocrine cells. This process may be more successful in vivo, or other molecular differences between SCLCs and LUADs could play a role. Nevertheless, these carefully conducted ‘negative’ studies commendably illustrate that important details are missing about this transition, which is involved in a range of human cancers and is associated with poor prognosis (Balanis et al., 2019).

Finally, the molecular insights gained from Inoue et al. could be expanded on and used in the clinic to both test and develop new therapies. This could help identify which patients are most at risk of tumor lineage switches and enable early interventions that improve the outcomes of treatment.

## References

[bib1] Aggarwal R, Huang J, Alumkal JJ, Zhang L, Feng FY, Thomas GV, Weinstein AS, Friedl V, Zhang C, Witte ON, Lloyd P, Gleave M, Evans CP, Youngren J, Beer TM, Rettig M, Wong CK, True L, Foye A, Playdle D, Ryan CJ, Lara P, Chi KN, Uzunangelov V, Sokolov A, Newton Y, Beltran H, Demichelis F, Rubin MA, Stuart JM, Small EJ (2018). Clinical and genomic characterization of treatment-emergent small-cell neuroendocrine prostate cancer: A multi-institutional prospective study. Journal of Clinical Oncology.

[bib2] Balanis NG, Sheu KM, Esedebe FN, Patel SJ, Smith BA, Park JW, Alhani S, Gomperts BN, Huang J, Witte ON, Graeber TG (2019). Pan-cancer convergence to a small-cell neuroendocrine phenotype that shares susceptibilities with hematological malignancies. Cancer Cell.

[bib3] Chen HJ, Poran A, Unni AM, Huang SX, Elemento O, Snoeck H-W, Varmus H (2019). Generation of pulmonary neuroendocrine cells and SCLC-like tumors from human embryonic stem cells. Journal of Experimental Medicine.

[bib4] Hanahan D, Weinberg RA (2011). Hallmarks of cancer: The next generation. Cell.

[bib5] Inoue Y, Nikolic A, Farnsworth D, Shi R, Johnson FD, Liu A, Ladanyi M, Somwar R, Gallo M, Lockwood WW (2021). Extracellular signal-regulated kinase mediates chromatin rewiring and lineage transformation in lung cancer. eLife.

[bib6] Ku SY, Rosario S, Wang Y, Mu P, Seshadri M, Goodrich ZW, Goodrich MM, Labbé DP, Gomez EC, Wang J, Long HW, Xu B, Brown M, Loda M, Sawyers CL, Ellis L, Goodrich DW (2017). Rb1 and Trp53 cooperate to suppress prostate cancer lineage plasticity, metastasis, and antiandrogen resistance. Science.

[bib7] Le Magnen C, Shen MM, Abate-Shen C (2018). Lineage plasticity in cancer progression and treatment. Annual Review of Cancer Biology.

[bib8] Marjanovic ND, Hofree M, Chan JE, Canner D, Wu K, Trakala M, Hartmann GG, Smith OC, Kim JY, Evans KV, Hudson A, Ashenberg O, Porter CBM, Bejnood A, Subramanian A, Pitter K, Yan Y, Delorey T, Phillips DR, Shah N, Chaudhary O, Tsankov A, Hollmann T, Rekhtman N, Massion PP, Poirier JT, Mazutis L, Li R, Lee JH, Amon A, Rudin CM, Jacks T, Regev A, Tammela T (2020). Emergence of a high-plasticity cell state during lung cancer evolution. Cancer Cell.

[bib9] Offin M, Chan JM, Tenet M, Rizvi HA, Shen R, Riely GJ, Rekhtman N, Daneshbod Y, Quintanal-Villalonga A, Penson A, Hellmann MD, Arcila ME, Ladanyi M, Pe'er D, Kris MG, Rudin CM, Yu HA (2019). Concurrent RB1 and TP53 alterations define a cubset of EGFR-mutant lung cancers at risk for histologic transformation and inferior clinical outcomes. Journal of Thoracic Oncology.

[bib10] Park JW, Lee JK, Sheu KM, Wang L, Balanis NG, Nguyen K, Smith BA, Cheng C, Tsai BL, Cheng D, Huang J, Kurdistani SK, Graeber TG, Witte ON (2018). Reprogramming normal human epithelial tissues to a common, lethal neuroendocrine cancer lineage. Science.

[bib11] Quintanal-Villalonga Á, Chan JM, Yu HA, Pe'er D, Sawyers CL, Sen T, Rudin CM (2020). Lineage plasticity in cancer: A shared pathway of therapeutic resistance. Nature Reviews. Clinical Oncology.

